# Sarcopenia and Frailty in Heart Failure: Is There a Biomarker Signature?

**DOI:** 10.1007/s11897-022-00575-w

**Published:** 2022-10-20

**Authors:** Ryosuke Sato, Mirela Vatic, Guilherme Wesley Peixoto da Fonseca, Stephan von Haehling

**Affiliations:** 1 Department of Cardiology and Pneumology, University of Göttingen Medical Center, Robert-Koch-Str. 40, 37075 Gottingen, Germany; 2 Heart Institute (InCor), University of São Paulo Medical School, Sao Paulo, SP Brazil; 3 German Center for Cardiovascular Research (DZHK), Partner Site Göttingen, Gottingen, Germany

**Keywords:** Heart failure, Sarcopenia, Frailty, Biomarkers

## Abstract

**Purpose of Review:**

Sarcopenia and frailty are common in patients with heart failure (HF) and are strongly associated with prognosis. This review aims to examine promising biomarkers that can guide physicians in identifying sarcopenia and frailty in HF.

**Recent Findings:**

Traditional biomarkers including C-reactive protein, aminotransaminase, myostatin, and urinary creatinine as well as novel biomarkers including microRNAs, suppression of tumorigenicity 2 (ST2), galectin-3, and procollagen type III N-terminal peptide may help in predicting the development of sarcopenia and frailty in HF patients. Among those biomarkers, aminotransferase, urinary creatinine, and ST2 predicted the prognosis in HF patients with sarcopenia and frailty.

**Summary:**

This review outlines the current knowledge of biomarkers that are considered promising for diagnosing sarcopenia and frailty in HF. The listed biomarkers might support the diagnosis, prognosis, and therapeutic decisions for sarcopenia and frailty in HF patients.

## Introduction

Heart failure (HF) is one of the leading global healthcare problems, afflicting an estimated 26 million people worldwide according to the current estimate [1]. The prevalence of HF increases with age, exceeding 10% in those aged 70 years old or older [2], and is expected to increase further due to the high proportion of elderly people in Western societies [3, 4]. As such, a better understanding of prevention strategies for HF and factors that exacerbate HF is essential.

Advanced stages of HF have been associated with a greater susceptibility to wasting syndromes like cardiac cachexia and sarcopenia [5]. Since both are usually associated with skeletal muscle loss, the clinical result can be physical frailty [6], a syndrome of increased vulnerability to the effects of stressors from age-related decline in the function and reserve of multiple physiological systems [7, 8]. As with HF, the prevalence of frailty is strongly associated with ageing [9], with 19–52% of outpatients and 56–76% of inpatients being affected by frailty according to a common frailty assessment method [10]. Thus, frailty is very common among patients with HF, and the combination of frailty and HF is associated with greater risk of exacerbation of symptoms, hospitalization, and death [11, 12]. Like physical frailty, sarcopenia is a geriatric syndrome characterized by age-related loss of skeletal muscle mass and muscle strength [13], often associated with chronic HF [14]. The results of the Studies Investigating Co-morbidities Aggravating HF (SICA-HF) have shown that the prevalence of sarcopenia in patients with chronic HF reaches 20% [15]. Loss of skeletal muscle mass in HF patients develops early, irrespective of left ventricular ejection fraction, and the two conditions accelerate each other and are closely associated with a decline in physical activity and poor prognosis [12, 16, 17]. As such, there is a growing emphasis on incorporating the assessment of sarcopenia and frailty into prognostic and therapeutic models of HF for more comprehensive management of HF patients [18, 19], and the latest HF guidelines of the European Society of Cardiology (ESC) dedicate a chapter to cachexia, sarcopenia, and frailty [2]. While cachexia can be easily diagnosed using weighing scales [20], the diagnosis of sarcopenia can only be reached using sophisticated assessment tools like dual-energy X-ray absorptiometry (DEXA), magnetic resonance imaging (MRI), or computed tomography (CT) [21]. Therefore, clinical researchers have called for biomarker assessment to detect muscle wasting and thus physical frailty early and more easily. Indeed, biomarkers are valuable tools for early and objective diagnosis and monitoring of various diseases and their severity [22, 23]. The purpose of this review is to summarize the current state of knowledge on promising biomarkers for the assessment of sarcopenia and frailty in HF.

## Diagnosis of Sarcopenia and Frailty

**Fig. 1:**
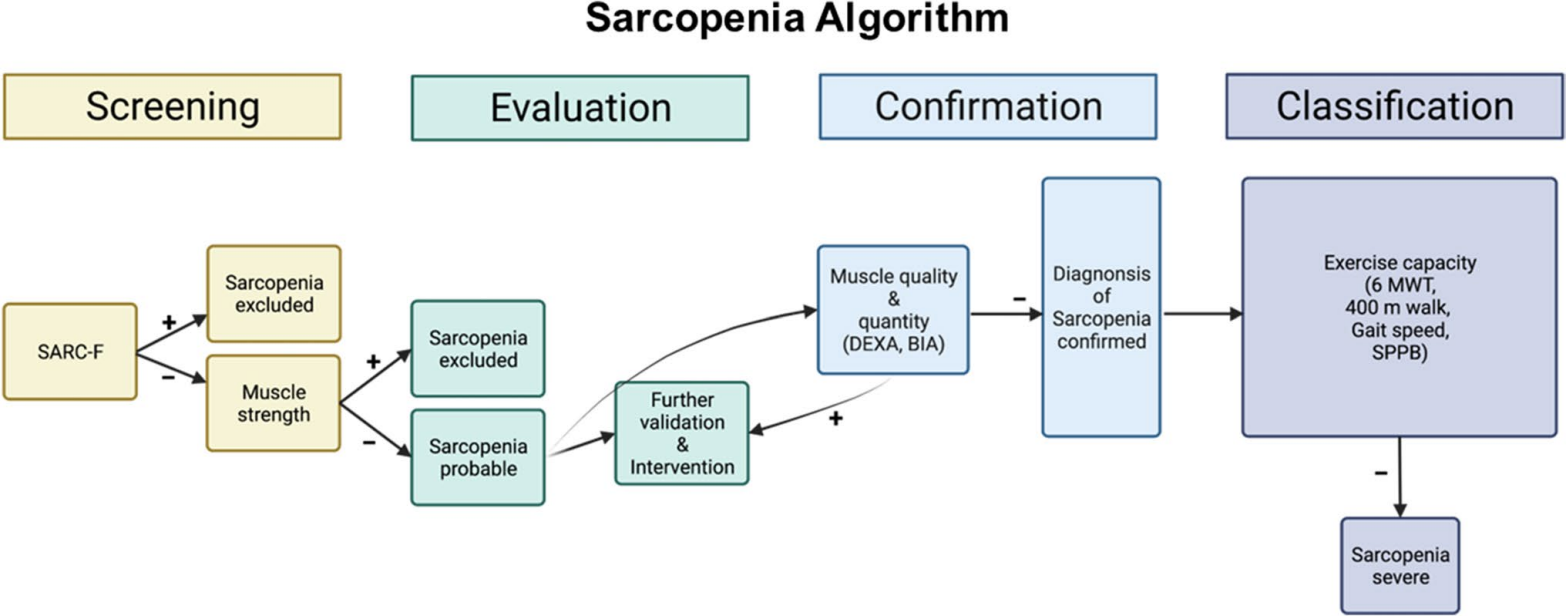
Sarcopenia: EWGSOP2 algorithm for case-finding, making a diagnosis and quantifying severity in practice. DEXA, dual-energy X-ray absorptiometry; BIA, bioimpedance analysis; 6MWT, 6-min walking test; SPPB, short physical performance battery. Modified from Cruz-Jentoft AJ et al. Age Ageing 2019

Sarcopenia and frailty are two entities that have been extensively discussed, from both clinical and preclinical perspectives. The prevalence of sarcopenia in elderly people has been reported to be 1–30% [21]. Back in 1988, a concept of sarcopenia was proposed to describe age-related muscle failure (from the Greek words: *sarx* for flesh and *penia* for deficiency) [24]. One decade later, Baumgartner and colleagues defined sarcopenia as an appendicular skeletal muscle mass (ASM, kg) per height in meters squared (m^2^) two standard deviations below the average of a healthy reference group, as measured by DEXA [25]. Upon a consensus conference led by the Society of Sarcopenia, Cachexia and Wasting Disorders (SCWD), the definition has been updated to “Sarcopenia, i.e., reduced muscle mass, with limited mobility,” is defined as an “individual with muscle loss whose speed of walking is ≤ 1 m/s or who cannot reach 400 m of walk during a 6-min walk, and who has an ASM corrected for height squared of 2 standard deviations or more below the average of healthy individuals between 20 and 30 years of age of the same ethnic group” [26]. In 2019, the European consensus definition has been revised by the European Working Group on Sarcopenia in Older People (EWGSOP) [21]. The updated sarcopenia operational definition uses the SARC-F questionnaire and reduced muscle strength as the fundamental parameters of sarcopenia. The revised consensus definition provides clear cutoff points for diagnostic variables of sarcopenia: ASM/height^2^ < 7.0 kg/m^2^ for men and ASM/height^2^ < 5.5 kg/m^2^ for women [21]. Furthermore, EWGSOP suggested the sarcopenia algorithm for case-finding, assessing a clinical suspicion, confirming a diagnosis and quantifying severity of sarcopenia in clinical practice (Fig. 1).

Next to muscle wasting, frailty is considered an important parameter of late-life health. The prevalence of frailty in community-dwelling adults aged 65 and older ranges extremely from 4 to 59% [27]. Frailty is a complex clinical syndrome that outlines a decline in physiological compensatory mechanisms, which leads to vulnerability, limited mobility, dependency, risk of falls and an increased morbidity, hospitalization rate, and mortality [28]. In the absence of a gold standard, two different operational models of frailty have been suggested: the “frailty phenotype,” proposed by Fried et al. [7] and the “frailty index,” proposed by Rockwood and Mitnitski [29]. A uni-dimensional construct, mainly focused on physical frailty, “frailty phenotype,” is characterized by the presence of at least three of the following parameters: (1) shrinking: unintentional weight loss of ≥ 4.5 kg in the previous 12 months or, at follow-up, loss of ≥ 5% of body weight in the previous year; (2) weakness: reduced grip strength in the lowest 20% at baseline, adjusted for gender and body mass index; (3) poor endurance and energy: as indicated by self-reported exhaustion and reduced peak oxygen uptake (peak VO_2_); (4) slowness: based on the 4-m gait speed test, adjusted for gender and height; (5) low physical activity level: a weighted score of kilocalories expended per week, adjusted for gender [7]. Contrary to Fried’s frailty phenotype, Rockwood’s “frailty index” represents a multi-dimensional construct related to accumulation of deficits over the course of time [29]. This concept encompasses physical, mental, nutritional, and socioeconomic frailties. More recently, the innovative tools composed of basic (BADL) and instrumental activities of daily life (IADL) have been shown to be useful for stratifying the mortality risk in frail elderly people [30].

Despite of the numerous definitions of frailty and sarcopenia, a proper diagnosis of these clinical conditions remains challenging. Therefore, evaluation of novel biomarkers of sarcopenia and frailty could be a very feasible approach.

## Mechanisms of Sarcopenia and Frailty in HF

The pathophysiology of sarcopenia and frailty involves metabolic changes, particularly increased catabolism [31]. Multiple pathophysiological mechanisms including malnutrition and anorexia, physical inactivity, hormonal changes, inflammation, oxidative stress, and insulin resistance lead to an increase in muscle protein catabolism, resulting in qualitative and quantitative muscle loss [31, 32]. This pathophysiologic phenomenon contributes to cardiometabolic and functional abnormalities in patients with HF, but it also appears to be exacerbated by HF [15]. The following overview provides possible mechanisms of sarcopenia and frailty in the context of HF.

### Malnutrition

An increased resting energy expenditure has been shown in HF, and the negative balance between energy demand and consumption leads to accelerated catabolism, resulting in protein-energy malnutrition [33, 34]. Patients with HF frequently develop nausea, gastroenteropathy, and ultimately anorexia and malabsorption caused by pulmonary and gastrointestinal oedema [32, 35]. Furthermore, several medications used for HF, such as digoxin, angiotensin-converting enzyme inhibitors, and β-blockers, can potentially contribute to anorexia [32, 36]. Diuretics may also contribute to trace element depletion through urination [32]. These conditions would naturally cause weight loss, reduced muscle strength, and endurance, leading to the development of frailty [37].

### Physical Inactivity

Reduced cardiac output and systemic congestion cause decreased dietary intake and exercise capacity in patients with HF [38]. Prolonged bed rest is often present in patients with HF. In the elderly, this situation also causes decreased insulin sensitivity, which further adversely affects muscle metabolism [39, 40]. Moreover, physical inactivity can diminish the muscle protein synthesis by impairing mammalian target of rapamycin (mTOR) signaling and amino acid transporter protein mass [41]. The linkage of these factors in muscle tissue results in a decline of skeletal muscle growth factor and an increase in oxidative damage, resulting in an imbalance between muscle protein synthesis and degradation that leads to skeletal muscle loss [38].

### Hormonal Changes

Insulin-like growth factor-1 (IGF-1) is an important ligand for growth hormone (GH) to exert its physiological effects, and decreased GH and IGF-1 levels are associated with impaired physical performance and sarcopenia [42, 43]. In an animal study in rats, Brioche et al. demonstrated that GH administration mitigates their sarcopenia from improvements in both muscle protein synthesis and mitochondrial biosynthesis [44]. In patients with HF, lower levels of GH and IGF-1 have been demonstrated when compared with healthy age-matched controls and these were associated with impaired cardiac performance and exercise capacity [45]. Furthermore, one randomized controlled trial has shown that long-term GH replacement therapy in patients with chronic HF improved peak VO_2_, left ventricular end-systolic volume, and left ventricular ejection fraction (LVEF) [45]. Testosterone levels decrease with age and have been reported to be associated with declines in muscle mass and strength in the elderly [46]. This point is important, because low testosterone levels are also common in patients with HF [47] and contribute to the progression of cardiac dysfunction through altered peripheral vascular resistance, increased cardiac afterload, and decreased cardiac output [48], leading to worse prognosis [49, 50]. Jankowska et al. reported that low testosterone levels are associated with lower peak VO_2_ in male patients with chronic HF [51]. Furthermore, several studies have reported that testosterone administration to patients with chronic HF improves peak VO_2_, walking distance, and muscle strength [52, 53]. Therefore, these hormonal changes may contribute to the progression of frailty in HF.

### Inflammation

Inflammation contributes to muscle wasting while it promotes cardiac dysfunction/remodeling and aggravates HF [54, 55]. There is a variety of mechanisms by which inflammation affects muscle metabolism. Tumor necrosis factor (TNF) induces apoptosis of myonuclei and stimulates local synthesis of other proinflammatory cytokines [56]. The transcription factor nuclear factor kappa B (NFkB) is activated by TNF and inhibits skeletal muscle differentiation by repressing MyoD mRNA at the post-transcriptional level [57]. TNF-like weak inducer of apoptosis decreases mitochondrial content and oxidative phosphorylation and inhibits angiogenesis in skeletal muscle [58]. Elevated levels of these inflammatory cytokines play an important role in the development of sarcopenia. Patients with HF have chronic low-level systemic inflammation and are reported to have elevated levels of inflammatory biomarkers such as TNF, C-reactive protein (CRP), and interleukin-6, which have been implicated in the loss of muscle mass and strength [59, 60]. Thus, systemic inflammation in HF contributes not only to the pathological progression of HF itself, but also to the development of skeletal muscle loss and dysfunction.

### Oxidative Stress

Oxidative stress occurs when there is an imbalance between the production of reactive oxygen species (ROS) and antioxidant defenses. ROS production increases with age and is considered one of the factors that contribute to senescence [61]. ROS accumulate during muscle contractile activity while the muscle enzyme scavenger system of muscle declines with age [62]. Excessive production of ROS contributes to reduced mitochondrial function and oxidative capacity [63] and accelerates skeletal muscle damage and degeneration [64]. Various oxidative stress markers have been reported to be elevated in patients with chronic HF, correlating with lower antioxidant levels and disease severity, as well as reduced exercise tolerance as expressed by the decline in peak VO_2_ [65, 66]. The possible mechanisms of oxidative stress-induced exacerbation of HF include hypertrophy, apoptosis/cell death and intracellular Ca^2+^ overload in cardiac myocytes, and endothelial dysfunction [67, 68]. In patients with HF, oxidative stress therefore may play an important role in the development of sarcopenia and exacerbation of HF itself.

### Insulin Resistance

Insulin can promote muscle protein synthesis by increasing muscle blood flow, amino acid delivery, and availability [69]. On the other hand, a decrease in muscle mass and a concomitant increase in intramuscular fat mass impair insulin-mediated glucose utilization, thus causing insulin resistance [70, 71]. Patients with HF have a strong insulin resistance due to a variety of factors, including inflammation, oxidative stress, inactivity, dysregulated secretion of adipokine/cytokine secretion, and increased renin-angiotensin II-aldosterone system activity and sympathetic nervous system [72]. Doehner et al. found that myofibrillar contractile function of the quadriceps muscle was positively correlated with insulin sensitivity in patients with chronic HF and healthy controls [73]. These findings indicate an association between insulin resistance and HF-related skeletal muscle wasting.

## Biomarkers of Frailty and Sarcopenia in HF

Biomarkers have emerged as indispensable and essential tools for the early diagnosis and for the monitoring of various diseases and their severity [22]. Optimal biomarkers support diagnostic as well as prognostic assessments and treatment decisions and help to stratify patients at risk who could benefit from preventive interventions [23]. Therefore, if appropriate biomarkers were available to identify sarcopenia and frailty in HF, early recognition and therapeutic intervention for these conditions could be possible. The following is a list of biomarkers associated with sarcopenia and frailty in HF patients that have been reported, but it cannot be regarded as complete because the field of biomarker development is vast and moving extremely fast (Table 1).

**Table 1:** Overview of the biomarkers in frailty and sarcopenia in HF

Marker	Category	Nature	Regulation in sarcopenia and frailty in HF	References
MicroRNAs	Inflammation	RNAs	Up/downregulated	[74•]
ST2	Inflammation	Cytokine	Upregulated	[75]
CRP	Inflammation	Protein	Upregulated	[76, 77]
Gal-3	Inflammation Fibrosis	Protein	Upregulated	[78]
ALT	Muscle remodeling Malnutrition	Enzyme	Downregulated	[79]
AAR	Fibrosis Malnutrition	Enzyme	Upregulated	[80, 81•]
Myostatin	Negative regulator of muscle growth Inflammation	Cytokine	Downregulated	[82]
P3NP	Muscle remodeling Fibrosis	Peptide	Up/downregulated	[83]
Urinary creatinine	Muscle remodeling	Waste product from muscle	Downregulated	[84•]

*AAR* aminotransferase ratio, *ALT* alanine transaminase, *CRP* C-reactive protein, *Gal-3* galectin-3, *HF* heart failure, *P3NP* procollagen type III N-terminal peptide, *ST2* suppression of tumorigenicity 2

### MicroRNAs

MicroRNAs (miRNAs) are short, non-coding RNAs that regulate gene transcription by repressing translation and degradation of mRNAs [85]. Skeletal muscle and other tissue-derived miRNAs are readily detectable in the circulation [74•], and aberrant expression of miRNAs has been associated with several skeletal muscle diseases, including sarcopenia [86]. Therefore, miRNAs have been proposed as candidates for early detection of sarcopenia, and many studies elucidated the potential role of miRNAs, including miRNA-1, miRNA-20a, miRNA-21, miRNA-34a, miRNA-146a, miRNA-185, and miRNA-223 as biomarkers of frailty [86, 87]. Patients with chronic HF have also been reported to show marked changes in their plasma miRNAs profiles. miRNA.155, miRNA.22, and miRNA.133 were proposed as promising biomarkers for the development and diagnosis as well as for prognostic assessments in HF [88].

Recently, Qaisar et al. investigated the association between circulating levels of specific miRNAs (miRNA-21, miRNA-434-3p, miRNA424-5p, miRNA-133a, miRNA-455-3p, miRNA-181a) and indices of sarcopenia during healthy ageing and in patients with chronic HF [74•]. Among these miRNAs, miRNA-133a, miRNA-434-3p, and miRNA-455-3p correlated most strongly with ASM index, and miRNA-434-3p also had the highest area under the curve in the testing of sensitivity and specificity for chronic HF diagnosis. These reports suggest that circulating specific miRNAs might be useful in determining frailty in HF patients; however, reproducibility of miRNA testing has been a major concern in recent years [89].

### ST2

Suppression of tumorigenicity 2 (ST2) is a member of the interleukin-1 family of proteins, and its role has been established as a surrogate marker of inflammation [90, 91]. Recently, several studies implicate this molecule in age-related diseases, suggesting that ST2 could be a potential candidate as a frailty biomarker [92, 93]. On the other hand, soluble ST2 is a potential pathophysiological mediator of myocardial hypertrophy and fibrosis [94]. The soluble form of ST2 is a decoy receptor that blocks the cardioprotective effects of interleukin-33 (IL-33) [91], and the presence of high levels of ST2 interaction, thereby inhibiting the activation of the cascade triggered by the IL-33/ST2 ligand interaction, leading to increased adverse cardiac remodeling of myocardial fibers, cardiac dysfunction, and worse cardiovascular outcomes [90]. Several studies have confirmed that ST2 levels are significantly elevated in HF patients and are associated with disease severity and prognosis [95, 96]. Pacho et al. assessed the value of early post-discharge circulating levels of several biomarkers for predicting short- and long-term outcomes in frail comorbid elderly patients admitted for HF, demonstrating that ST2 was a significant predictive biomarker for short-term HF-related rehospitalization and all-cause death, as well as for long-term HF-related rehospitalization [75]. These findings suggest that ST2 might be a potential predictive biomarker in frail HF patients.

### CRP

CRP is a marker of acute inflammation and mainly formed as an acute phase reactant by the liver. A vast array of studies has shown that elevated serum CRP levels are associated with sarcopenia and frailty [97–99]. Several reports have also reported that CRP is associated with disease severity in HF patients. The Val-HeFT study showed that elevated CRP levels are associated with more severe signs of HF, like New York Heart Association (NYHA) class III/IV and lower LVEF [100]. Minami et al. reported that markedly elevated CRP levels at admission in patients with acute HF are associated with greater all-cause mortality [101]. The relationship between frailty and CRP levels in HF patients is also of interest. Boxer et al. found that CRP levels are negatively correlated with the 6-min walk distance in HF patients with LVEF less than 40% [76]. Ribeiro et al. reported that frailty, assessed by the physical and multidimensional approach, was significantly associated with only high sensitivity CRP among several inflammatory and humoral biomarkers in outpatients with HF aged ≥ 60 years [77]. These findings suggest that CRP is a promising sarcopenia and frailty biomarker in HF patients, and further studies should seriously consider inflammation as a possible pathophysiological pathway for frailty in HF patients.

### Galectin-3

Galectin-3 (Gal-3), a β-galactoside-binding lectin whose levels increase with ageing, plays a significant role in systemic inflammation, fibrosis, atherosclerosis, and HF progression [102, 103]. Elevated Gal-3 levels are associated with adverse outcomes such as cardiovascular disease as well as infections, liver fibrosis, and cancer [104]. Testa et al. reported that adding Gal-3 to B-type natriuretic peptide (BNP) values significantly improved the predictive power for mortality in elderly patients with chronic HF, as well as higher levels of disability [105]. In addition, the inhibition of Gal-3 has been reported to prevent adverse cardiac remodeling by interfering with myocardial fibrosis in an experiment with HF mice [106]. Various studies have also reported that cellular senescence is regulated by the Gal-3 signaling pathway. [107, 108]. Komici et al. investigated the potential of Gal-3 to serve as a biomarker of frailty in elderly HF patients with reduced ejection fraction, showing that serum Gal-3 levels were significantly associated with Clinical Frailty Scale and, furthermore, adding serum Gal-3 to the prognostic model improved the net clinical benefit [78]. Gal-3 may be a useful biomarker for detecting various comorbidities including frailty and predicting prognosis in elderly HF patients.

### Aminotransferase

Alanine transaminase (ALT), which is abundant in the liver and facilitates the conversion of pyruvate to the amino acid alanine, is an accessible and inexpensive biochemical assay that primarily monitors cellular and hepatic damage in the clinical setting. On the other hand, when the liver parenchyma is intact, ALT plasma levels are a good marker of systemic skeletal muscle mass and muscle strength [109]. Low ALT has also been reported to be a surrogate marker of malnutrition [80], and numerous studies accumulated the evidence that lower ALT levels may be reliable markers of sarcopenia and frailty in a variety of populations [110, 111]. Recently, Segev et al. reported that in patients hospitalized for HF, the low ALT group had a higher incidence of cerebrovascular disease, dementia, and malignancy causing frailty and sarcopenia and a significantly higher all-cause mortality rate than those with high ALT [79].

Besides, liver fibrosis associated with HF, a condition known as cardiohepatic syndrome, is a strong predictor in patients with HF [112]. The aspartate aminotransferase to alanine aminotransferase ratio (AAR) is one such fibrosis marker, and it has been reported that there is a significant association between high AAR and low body mass index, malnutrition, and poor prognosis in patients with acute HF [80]. Maeda et al. demonstrated that high AAR was associated with poor physical function as assessed by short physical performance battery (SPPB) and 6-min walk distance and was an independent predictor of all-cause death in elderly patients hospitalized for HF [81•]. Thus, aminotransferase plasma levels, biomarkers of sarcopenia and frailty, can also be useful for their detection and risk stratification in HF patients.

### Myostatin

Myostatin, a member of the transforming growth factor beta family, also known as growth differentiation factor 8, is expressed primarily in skeletal muscle and negatively regulates muscle mass [113], and its gene and protein expression have been demonstrated to be increased in older men compared with younger individuals [114]. Myostatin is to some extent also expressed in cardiac muscle, where it also exhibits fibrosis-promoting properties [113]. It has been shown that serum myostatin levels in patients with HF are significantly elevated compared to healthy controls [115], and in lateral vastus muscle biopsies, Gielen et al. found that baseline myostatin mRNA expression was about 50% higher in patients with chronic HF compared to that in age-matched healthy controls [116]. Furthermore, Heineke et al. reported that myostatin released from cardiomyocytes causes skeletal muscle atrophy in a chronic HF mouse model [82]. In one experiment with a myostatin-inhibited mouse model, physical function and whole-body metabolism also reported to be significantly improved in aged mice [117]. These findings suggest that myostatin is not only involved in HF-related muscle wasting, but that it may also be a therapeutic target in frail HF patients.

### P3NP

Procollagen type III N-terminal peptide (P3NP), a fragment released into the circulation when procollagen type III is cleaved to produce collagen type III, is proposed as a novel biomarker of muscle remodeling [118]. In specific hormonal therapies [118] and resistance training [119], plasma P3NP has been associated with changes in skeletal muscle mass. On the other hand, P3NP is the primary collagen type contributing to cardiac fibrosis and is one of the few biomarkers that reflects the severity of myocardial fibrosis [120]. P3NP is elevated in patients with advanced HF [121], and several studies have reported that it responds to therapies such as angiotensin-converting enzyme inhibitors and aldosterone antagonists [122, 123]. More recently, Qaisar et al. reported that a cumulative risk score calculated from a battery of biomarkers including P3NP was effective in identifying high-risk groups for sarcopenia in patients with HF [83]. P3NP may be of potential values for early diagnosis and evaluation of sarcopenia in HF patients.

### Urinary Creatinine

Twenty-four-hour urinary creatinine excretion has been suggested as a non-invasive, an inexpensive and easily accessible biomarker to evaluate muscle wasting and cachexia, as it is produced by the stable conversion of creatine, which is abundant in skeletal muscle [124]. The reliability of 24-h urinary creatinine excretion as an indicator of muscle wasting has been examined in various chronic disease cohorts, and in chronic HF, lower urinary creatinine was shown to be an independent predictor of major adverse cardiovascular events and all-cause mortality [125]. Pandhi et al. also examined the association between spot urinary creatinine and changes in body composition and outcomes in the BIOSTAT-CHF trial [84•]; lower spot urinary creatinine levels were associated not only with weight loss, decreased exercise capacity and renal dysfunction, but also with the severity of HF, HF rehospitalizations, and all-cause mortality. Moreover, several studies suggest that proteins, amino acids, and creatine supplementation in adding to resistance training can improve skeletal muscle mass and muscle function in elderly populations [126, 127]. Urinary creatinine may therefore be an easily accessible biomarker for risk stratification of frailty in HF patients as well as a target for therapeutic intervention in HF patients with frailty.

## Conclusions

The rapid ageing of the population has led to a worldwide increase in the prevalence of HF. The prevalence of the geriatric syndromes sarcopenia and frailty is also on the rise. The pathogenesis of these diseases largely overlaps, and their combination exacerbates each other and significantly worsen prognosis, emphasizing the importance of early detection and intervention. While assessing the biomarkers outlined in this review is a promising way to evaluate sarcopenia and frailty in HF, some of these biomarkers are nonspecific and only able to capture single aspects of the diseases. Further progress in the field and more comprehensive approach are essential for the prevention, risk stratification, intervention, and improvement of sarcopenia and frailty in patients with HF.
